# Prevalence of* Helicobacter pylori vacA* Genotypes and* cagA* Gene in Dental Plaque of Asymptomatic Mexican Children

**DOI:** 10.1155/2017/4923640

**Published:** 2017-11-01

**Authors:** Alejandra Mendoza-Cantú, Víctor Hugo Urrutia-Baca, Cynthia Sofía Urbina-Ríos, Myriam Angélica De la Garza-Ramos, Martha Elena García-Martínez, Hilda H. H. Torre-Martínez

**Affiliations:** ^1^Posgrado de Odontopediatría, Facultad de Odontología (FO), Universidad Autónoma de Nuevo León (UANL), Av. Dr. Aguirre Pequeño 1504, Col. Mitras Centro, 64460 Monterrey, NL, Mexico; ^2^Departamento de Microbiología e Inmunología, Laboratorio de Inmunología y Virología (LIV), Facultad de Ciencias Biológicas (FCB), Universidad Autónoma de Nuevo León (UANL), Pedro de Alba y Manuel L, Barragán S/N, Cd. Universitaria, 66450 San Nicolás de los Garza, NL, Mexico; ^3^Unidad de Odontología Integral y Especialidades (UOIE), Facultad de Odontología (FO) and Centro de Investigación y Desarrollo en Ciencias de la Salud (CIDICS), Universidad Autónoma de Nuevo León (UANL), Av. Dr. Aguirre Pequeño y Silao S/N, Col. Mitras Centro, 64460 Monterrey, NL, Mexico; ^4^Posgrado de Ortodoncia, Facultad de Odontología (FO), Universidad Autónoma de Nuevo León (UANL), Av. Dr. Aguirre Pequeño 1504, Col. Mitras Centro, 64460 Monterrey, NL, Mexico

## Abstract

The variability in* Helicobacter pylori vacA* and* cagA* genes has been related to the progression of the gastrointestinal disease; also the presence of* H. pylori* in the oral cavity has been associated with periodontal disease in adults, but, in children without dyspeptic symptoms, little is known about this. We evaluated the prevalence of* H. pylori* and the presence of* vacA*/*cagA *genotypes in the oral cavity of Mexican children without dyspeptic symptoms. The gingival status was measured, and dental plaque samples (*n* = 100) were taken. 38% of children were positive for* H. pylori* 16S rRNA gene by qPCR. A significant association between* H. pylori* oral infection and gingival status was observed (*P* < 0.001). In 34.6% (9/26) of mild gingivitis cases,* s1m2* genotype was found, while* s1m1* was typed in 50% (3/6) of moderate gingivitis. The* cagA* prevalence among* H. pylori*-positive children was 80.8% (21/26), 83.3% (5/6), and 16.7% (1/6) of cases of mild gingivitis, moderate gingivitis, and nongingivitis, respectively (*P* < 0.001). The* s1m1/cagA*+ combinational genotype was the most detected in children with gingivitis. Our results suggest that the prevalence of* H. pylori* and detection of* vacA/cagA* genotypes-associated gastrointestinal disease in the oral cavity could be related to the progression of gingivitis in asymptomatic children.

## 1. Introduction

*Helicobacter pylori* is a Gram-negative species which colonizes gastric mucosa in humans [1].* H. pylori* infection is associated with gastrointestinal tract diseases including chronic gastritis, peptic ulcer, mucosa-associated lymphoproliferative disorders, and gastric cancer [2–4]. The global prevalence of* H. pylori* infection is more than 50%. This prevalence may vary significantly within and among countries, according to geography, ethnicity, age, and socioeconomic factors [5, 6]. It has been reported that* H. pylori* infection rate is up to 70% in developing countries, while the rates in developed countries and regions, such as Australia and Western Europe, are only 25% and 28%, respectively [7]. Socioeconomic factors also explain a significant proportion of the difference in* H. pylori* prevalence. In the third National Health and Nutrition Examination Survey conducted in the United States, a 25% prevalence of* H. pylori* infection was found in children and young adults between 6 and 19 years. In the American population, prevalence was 42%; prevalence was higher in children of a low socioeconomic status, in those whose mothers had a lower education level, and in those living in crowded conditions [8]. O'Rourke et al. evaluated the* H. pylori* infection in Mexican and American children living on both sides of the Rio Grande (a river that is dividing both countries); a slightly higher prevalence was observed in Mexicans compared to Americans [9]. In a similar study by Goodman et al. they found a seroprevalence of 74% and 56% of Mexican and American women, respectively [10]. The relationship between socioeconomic status and* H. pylori* infection has been reported too in countries such as Bolivia [11].

Epidemiologic studies have shown evidence that most infections are acquired in childhood, but the specific age of acquisition and the factors associated with its persistence are not clear [12]. In developing countries, 70 to 90% of the population become infected during childhood; in developed countries, a smaller percentage (~10%) of children become infected, and the prevalence of infection increases with age [10]. In Mexico, a seroepidemiological national survey in 1998 found a national prevalence of 66% in the general population, 20% in children younger than one year, 50% in children younger than ten years, and 70% in adults younger than 20 years. There were differences in prevalence, depending on the economic development of the regions (86.1% prevalence in southeastern Mexico, 47.1% in the northeast) [13].

*H. pylori* infection mainly spreads through consumption of contaminated food and drinks or is transmitted orally. Several studies have evaluated the oral cavity, and* H. pylori* has been detected in dental plaque and saliva [14–16]. It is known that the presence of* H. pylori* in the oral cavity is one of the main causes of the reappearance of gastric* H. pylori* infection and that treatment of oral infection significantly increases eradication of* H. pylori* infection in the stomach [17]. Nevertheless, the role of dental plaque as an extragastric reservoir in* H. pylori* transmission is controversial [18–20]. Also, the oral* H. pylori* infection has been related to oral cavity problems, like gingivitis, periodontitis [21, 22], and recurrent aphthous stomatitis (RAS) [23]. Nisha et al. showed a highly significant association was found between periodontal disease and colonization of* H. pylori* in dental plaque [24].

*H. pylori* has many virulence factors that allow establishment, colonization, and damage to the gastric epithelium. An important virulence factor secreted by* H. pylori* is VacA cytotoxin, which induces vacuolization and cell death in epithelial cells. The encoding* vacA* gene is present in all* H. pylori* strains, but it is polymorphic, comprising variable signal regions (type* s1* or* s2*) and midregions (type* m1* or* m2*) [3, 26]. Strains with alleles* s1/m1* secrete an active toxin that is associated with the development of peptic ulcers and gastric cancer; also, this region was recently described as a determining factor in cytotoxicity and development of gastric disorders [27]. Some strains of* H. pylori* produce the CagA cytotoxin, as part of the* cag* pathogenicity island (PAI), a region of 40 kb DNA encoding about 31 genes that make up a type IV secretion system, which allows the release of oncoprotein CagA directly to the cytosol of gastric epithelial cells [28]. Upon release, CagA leads to dephosphorylation of cellular proteins, changes in morphology, increased cell proliferation, chronic inflammation, and carcinogenesis processes [29]. It is reported that individuals infected with* cagA*-positive and certain* cagA* alleles (e.g.,* cagA1a*) have a higher risk of developing the peptic ulcer and gastric cancer [30].

Recently, some studies have evaluated the prevalence of* H. pylori cagA* and* vacA* genes in saliva and dental plaque [31–33], but its frequency has not been extensively studied in Mexico.

In this study, we evaluated the prevalence of* H. pylori *and the presence of* vacA* genotypes and* cagA *gene in dental plaque of Mexican children without dyspeptic symptoms.

## 2. Materials and Methods

### 2.1. Patients

From November to December 2015, one hundred Mexican children (from 3 to 16 years old) from the Back2Back nonprofit organization at Monterrey city, Nuevo León, Mexico, were selected to participate in this study. The exclusion criteria were as follows: (1) current history of antibiotic usage or during the previous two months, (2) systemic disease, (3) dyspeptic symptoms, and (4) history of gastrointestinal diseases. Before selection, the informed consent form was obtained from guardians and authorities, who declared the willingness of children to allow the use of the samples and anonymous data for research purposes, and also this study was approved by The Research Bioethics Committee in Dental Science College at Universidad Autónoma de Nuevo León (UANL).

### 2.2. Measurement of Gingivitis

The modified gingival index (MGI), defined by Lobene et al., is a noninvasive method (no probing) [34]. The MGI was used to assess gingival inflammation in participants, as follows: (0) normal (no inflammation), (1) mild inflammation (slight change in color, little change in texture) of any portion of the gingival unit, (2) mild inflammation of the entire gingival unit, (3) moderate inflammation (moderate glazing, redness, edema, and/or hypertrophy) of the entire gingival unit, and (4) severe inflammation (marked redness and edema/hypertrophy, spontaneous bleeding, or ulceration) of the gingival unit. The clinical examination was performed by the same observer.

### 2.3. Sample Collection

Dental plaque was collected with a sterile bamboo skewer by a downward scrape against the vestibular surface (supragingival) of the superior first molar. After that, the sample was suspended in 1.5 ml of trypticase soy broth (TS; Becton Dickinson, Franklin Lakes, NJ) supplemented with 30% (w/v) glycerol for the maintenance of microbial viability and then storage at −70°C, until use.

### 2.4. DNA Extraction

Dental plaque samples were centrifuged at 12,000 ×g for 2 min, then washed with 0.01 m Phosphate-Buffered Saline (PBS; 0.1 m, pH 7.4), and suspended in 100 *μ*l Tris-EDTA buffer (TE; 10 mM Tris and 1 mM EDTA, at pH 7.4). For enzymatic cell lysis, 10 *μ*l of lysozyme (10 mg/ml) and 10 *μ*l of Proteinase K (10 mg/ml) were added and incubated at 56°C for 30 min. Total DNA was extracted using a High Pure PCR template preparation kit (Roche Diagnostics, GmbH, Mannheim, Germany) according to the manufacturer's recommendations and the DNA concentration was measured at 260 nm in a spectrophotometer (NanoDrop 8000 UV-Vis; Thermo Scientific, Wilmington, DE). The DNA samples were stored at −20°C, until use.

### 2.5. Detection of* H. pylori* and* cagA* Gene by qPCR

The presence of* H. pylori* and* cagA* status were assessed by qPCR using primers/probe for 16s rRNA gene and* cagA* gene, respectively (Table 1). The qPCR primers/probes were designed by IDT PrimerQuest tool (http://www.idtdna.com/primerquest/), DNA sequences were obtained from the GenBank database (https://www.ncbi.nlm.nih.gov/genbank/), and BLAST search (http://blast.ncbi.nlm.nih.gov/) was performed to check the specificity of the primers/probe sequences. The efficiency of primer pairs and probes was evaluated by performing serial dilutions from 0.1 to 100 ng of DNA, and the value of the slope was obtained by simple linear regression analysis (*R*^2^); the primers and probes showed ≥95% efficiency values. The qPCR reactions were performed in a 96-well plates containing 12.5 *μ*L of 2x maxima probe qPCR master mix (Thermo Scientific, Carlsbad, California, USA), 0.3 *μ*M primers mix forward/reverse, 0.2 *μ*M probe, and 100 ng of the DNA sample, in free-nucleases water to a final volume of 25 *μ*L; 100 ng of DNA from* H. pylori* ATCC 700824 or ATCC43504 strains and nuclease-free water were added as positive and negative control, respectively. The qPCR assay was carried out in a LightCycler 480II thermocycler (Roche, Mannheim, Germany). The thermocycler was programmed with a monocolor hydrolysis probe format (6-FAM, filter combination 465–510) as follows: one denaturalization cycle (95°C, 10 min), thirty-five amplification cycles (95°C, 10 s, 4°C/s ramp rate; 55°C, 15 s, 2°C/s ramp rate; 72°C, 15 s, 4°C/s quantification analysis ramp rate), one melting cycle (95°C, 5 s, 4°C/s ramp rate; 65°C, 1 min, 2.2°C/s ramp rate, 97°C with a 5°C continuous acquisition), and one cooling cycle (40°C, 10 s, 1.5°C/s ramp rate).

### 2.6. Detection of* H. pylori vacA* Alleles

The* vacA* gene and “*s*” and “*m*” region genotyping was performed by nested PCR using a set of oligonucleotides for each gene fragment, as previously described by Koehler et al. [25]. For the signal region* (s)* detection, vacA1F/vacA1R and vacA2F/vacA1R oligonucleotides were used in the first and the second amplification rounds, respectively. For the middle region 1* (m1)* allele detection in the first round m1F1/m1R1 primers were used; for the second round, m1F2 and m1R2 were used. For middle region 2* (m2)*, m2F1/m2R1 and m2F2/m2R2 primers were employed in the first and the second runs, respectively (Table 1).

**Figure 1:**
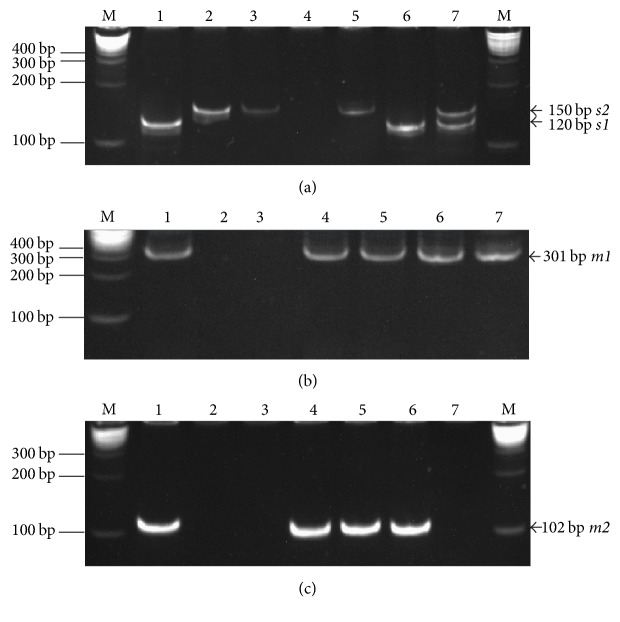
Results of the gel electrophoresis for identification of* H. pylori vacA* alleles and genotypes in dental plaque samples of Mexican children. (a) Detection of *s* alleles. Lane M, molecular weight marker 0.1 to 10 kb (BioLabs Inc.); lane 1, positive control (DNA from* H. pylori* ATCC 43504* s1m1* genotype); lanes 2-3, positive control (DNA from gastric biopsy with* H. pylori s2m2 *genotype); lane 4, negative control (without DNA); lane 5,* s2* allele positive (DNA from sample); lane 6,* s1* allele positive (DNA from sample); lane 7,* s1* and* s2* alleles positive (DNA from sample). (b) Detection of* m1* allele. Lane M, molecular weight marker 0.1 to 10 kb (BioLabs Inc.); lane 1, positive control (DNA from* H. pylori* ATCC 43504* s1m1* genotype); lane 2, negative control (without DNA); lane 3,* m1 *allele negative (DNA from sample); lanes 4–7,* m1* allele positive (DNA from sample). (c) Detection of* m2* alleles. Lane M, molecular weight marker 0.1 to 10 kb (BioLabs Inc.); lane 1, positive control (DNA from gastric biopsy with* H. pylori vacA s2m2* genotype); lane 2, negative control (without DNA); lanes 3 and 7,* m2* allele negative (DNA from sample); lanes 4–6,* m2* allele positive (DNA from sample).

All the PCR were prepared using 1x NH_4_ reaction buffer, 3 mM MgCl_2_, 10 *μ*M of each deoxynucleoside triphosphate, 0.2 *μ*M of each primer, 2.5 units Taq Polymerase (Bioline USA Inc., Taunton, USA), and 200 ng of DNA up to a final volume of 25 *μ*L. The PCR were carried out in MJ Mini Thermal Cycler (Bio-Rad Lab). The PCR program comprised 1 cycle of initial denaturation (95°C, 5 min), 35 cycles of denaturation (95°C, 1 min), annealing (58°C, 1 min) and extension (72°C, 1 min), and 1 cycle of final extension (72°C, 5 min). For the second amplification round, 1 *μ*l DNA template from the first PCR and 0.2 *μ*M of each primer were used. PCR was performed according to the PCR protocol described above. The PCR products were visualized on 8% (v/v) acrylamide gel (Bio-Rad Lab) in Tris-Borate-EDTA buffer (TBE; 40 mM Tris, 45 mM boric acid, 1 mM EDTA, at pH 7.4). The gel was stained with 1 *μ*g/mL of ethidium bromide solution and transferred to ultraviolet transilluminator (Gel Doc XR+ Imager; Bio-Rad Lab). The sizes of the amplicons were estimated by comparison with a DNA size marker (Quick-Load® 2-Log DNA Ladder; New England Biolabs, MA, USA). 120 bp and 150 bp PCR products corresponded to* s1* and* s2* alleles, respectively, while 301 bp and 102 bp bands corresponded to* m1* and* m2* alleles, respectively.* H. pylori* ATCC43504 strain was used as positive control (Figure 1).

### 2.7. Statistical Analysis

The association between* H. pylori* oral infection and each nominal variable were determined by the Chi-square and Fisher's exact tests; one-way ANOVA tests, post hoc Tukey tests, or Student's *t*-tests were used for numeric variables. The *P* < 0.05 value was considered statistically significant. The data were analyzed using SPSS IBM statistics software v22.0.

## 3. Results

### 3.1. The History of Participants and* H. pylori* Detection

This study was designed to determine the frequency of* H. pylori cagA* and* vacA* genes in dental plaque of children from the northeastern Mexico. A total of 100 children without gastric clinical manifestations, 50 males and 50 females ranging in age from 3 to 16 years with an average age of 8.95 ± 3.72, were included. Also, the participants were divided according to the gingival clinical presentation, 59 (59.0%) were diagnosed as no inflammation, 35 (35.0%) were diagnosed as mild inflammation, and 6 (6.0%) were regarded as moderate inflammation (Table 2). Among 41 gingivitis cases, 62.1% (22/35) of mild inflammation cases were male, while 83% (5/6) of moderate inflammation cases were female. A significant difference (*P* < 0.05) was observed between the mean age of patients with moderate inflammation (4.83 ± 2.04 years old) and those who did not present inflammation (9.47 ± 3.42 years old), as shown in Table 3.

The detection of* H. pylori* 16S rRNA gene by qPCR revealed that 38% of samples were positive. Among* H. pylori*-positive children, 55.3% (21/38) and 44.7% (17/38) were male and female, respectively. The mean age of* H. pylori*-positive individuals was 8.37 ± 4.17 years (Table 2).

### 3.2. Relationship between the* H. pylori* Oral Infection and the Gingival Clinical Presentation

A significant association between* H. pylori* oral detection and gingival status was observed (*P* < 0.001). 68.4% (26/38) of* H. pylori-*positive children shown mild inflammation, while 15.8% (6/38) showed no inflammation; all of the cases of moderate inflammation were positive (Table 2).

### 3.3. Frequency of* H. pylori vacA *Alleles and Association of Genotypes with the Gingival Clinical Presentation

The* vacA* allelic variants were determined in patients with dental plaque* H. pylori*-positive, as shown in Figure 1. The* vacA s1 *and* m1* alleles were most frequent for the signal and middle region, respectively. The* vacA s1m2* genotype was the most common among* H. pylori*-positive patients, with 28.9% (11/38), followed by* s1m1 *genotypes with 26.3% (10/38) (Table 4). The coinfection of* s1m1* with* s2m1* was observed in 13.2% (5/38) of* H. pylori*-positive samples. In 5.3% (2/38) of patients, the* s1* allele was detected, but the *m* region was undetectable* (s1m0)*; the* s2* allele was identified in 1 patient, but the *m* region could not be identified* (s2m0)*.

The prevalence of* vacA* genotypes and alleles varied with gingival status. The* s2m1 *genotype was detected in 50% (3/6) of* H. pylori*-positive children without gingivitis. In 34.6% (9/26) of mild inflammation cases,* s1m2* genotype was found, while* s1m1* allele combination was typed in 50% (3/6) of moderate inflammation (Table 4). The coinfection of* s1m1/s1m2* was observed only in patients with gingivitis (4 and 1 for mild and moderate inflammation, resp.).

### 3.4. Association between the Presence of* H. pylori cagA* Gene and Gingival Clinical Presentation

We found that the prevalence of* cagA* gene was 27% (27/100). In 71.1% (27/38) of* H. pylori*-positive samples,* cagA* gene was detected (Table 4). The* cagA* gene status has shown a significant relationship with gingival status (*P* < 0.001). Also, the* cagA* prevalence among* H. pylori*-positive children was 80.8% (21/26), 83.3% (5/6), and 16.7% (1/6) of cases of mild gingivitis, moderate gingivitis, and nongingivitis, respectively.

The combinational* s1m1/cagA*+ genotype was detected in 23.7% (9/38) of* H. pylori*-positive children. The same genotypic combination of* vacA/cagA* was the most frequent in children with gingivitis (5/26 and 3/6 of cases with mild and moderate gingivitis, resp.), while* s2m1*/*cagA*− combinational genotype was most prevalent in children without gingivitis (Table 4).

## 4. Discussion

It has been demonstrated that* H. pylori* infection is a risk factor for the development of gastric pathologies, including gastric cancer [4]. Several studies of* H. pylori* prevalence have shown the strong association with sociodemographic and socioeconomic factors where the developing countries are usually the most affected [8, 10–13]. The gastric infection is mainly acquired during the first years of life, where common symptoms are not present. Several studies have concluded that the high frequency of* H. pylori* could be due to its multiple mechanisms of transmission, where the oral cavity plays a determinant role as a source of transmission to other hosts and a focus for the continuous gastric reinfection after eradication therapy [14]. However, some researchers do not agree with this approach.

In this study, the detection of* H. pylori* and* cagA* gene was performed by qPCR. The use of TaqMan qPCR probes would allow accurate identification of* H. pylori* and would prevent the detection in the oral cavity of other microorganisms that are close phylogenetically to* H. pylori*, as* Campylobacter *species [35]. Our results show that 38% of dental plaque samples from children were* H. pylori*-positive. All participants were apparently healthy and did not report any gastric disease at the time of the evaluation (Table 2). Our results agree with those found by Duque et al. and those reported by Mendoza et al., both studies in Mexican school children, but these reports used urea breath test (UBT) for the diagnosis of infection [8, 36]. The agreement would be attributed to the fact that both diagnosis methods (UBT and PCR) have an estimated sensitivity of 75% to 100%. However, PCR has a higher specificity than UBT due to the presence of other urease-producing microorganisms in the stomach [37]. In 2014, Valdez-Gonzalez et al. evaluated the presence of* H. pylori *in dental plaques of 40 Mexican children using qPCR, and 35% of the samples were positive [16]. Our results are different from those reported by Castro-Muñoz et al.; they studied 162 asymptomatic Mexican children, and 13% of oral swab sample (from inside cheeks) were positive by PCR [15]. This difference may be due to the site from which samples were collected because the detection rates in dental plaques were more than those in other locations of oral cavity [14]. Zheng and Zhou evaluated the detection rate of* H. pylori* in subgingival and supragingival plaque samples from adults with/without periodontitis, and they observed a higher detection rate in subgingival plaques than that in supragingival plaques (*P* < 0.05) [22]. In our study, supragingival plaque samples were taken because it is a noninvasive sampling method (not probing) for children. It is possible that the microaerobic conditions and microbial associations in dental plaque could favor the persistence of* H. pylori* in the oral cavity, constituting an ideal site for sampling in* H. pylori* oral detection studies.

The presence of* H. pylori* in oral cavity would provide a foundation for a role in* H. pylori *transmission. Miyabayashi et al. investigated the effect of oral* H. pylori* on the stomach. The investigators observed that their patients with oral* H. pylori* were found to be at a significantly higher risk for gastric reinfection after having received adequate treatment. The authors also determined that drugs used for the eradication of the gastric* H. pylori *did not affect the oral* H. pylori* [38]. Most published studies have reported the oral cavity (dental plaque and saliva) as a source for subsequent gastric infections or reinfections in the same patient and as a reservoir for oral-oral transmission [19, 39]. However, some authors suggest that* H. pylori* is a microorganism transitory in the human oral cavity [40]. The mechanism by which* H. pylori* reaches the oral cavity is unknown. It is possible that the occasional reflux from the gastric reservoir allows colonization of the oral cavity. It is also possible that the reverse is true.

Our results indicate a significant frequency of* H. pylori* in dental plaque of asymptomatic children where the dental plaque would act as a reservoir for oral-oral dispersal to the population. However, our results cannot confirm the bacterial viability of oral* H. pylori* nor the ability to transmit to other individuals. This subject requires considerably more investigation due to the fact that the involvement of dental plaque in the transmission of* H. pylori *can be confirmed and that new measures can be tailored toward the prevention of oral spread.

It has been demonstrated that exposure to an* H. pylori*-positive family is a risk factor for persistence of infection in children under five years. Cervantes et al. followed a large cohort of children throughout the first years of life and concluded that when siblings are close in age, the older sibling may be an important source of* H. pylori* transmission for younger siblings [41]. In our study, we observed that 31.6% of* H. pylori*-positive subjects corresponded to the groups of age from 3 to 5 years (12/38) and 12 to 14 years (12/38). The children come from a care center for orphans where the coexistence between the older children and the younger ones would have favored the transmission among the children. However, this possibility could not be confirmed in this study. On the other hand, other studies have reported the familial transmission between parents and children [42, 43]. After many years of research, very little is known about the details of the modes of transmission of* H. pylori* and its propagation pathways. The primary modes of transmission are thought to be fecal-oral and oral-oral, but some indirect evidence has also been published for transmission via drinking water and other environmental sources [44].

Dental plaque harbors at least 400 different bacterial species and forms a biofilm in which organisms are intimately associated with each other and embedded in an exopolymeric matrix (salivary polymers and microbial extracellular products). This biofilm adheres to supragingival and subgingival tooth surfaces [19]. The accumulation of microorganisms and increased concentration of bacterial metabolites in gingiva induces an inflammatory process, called gingivitis. Gingivitis is one of the first clinical manifestations of the periodontal disease [45].* H. pylori* oral infection has been related to oral pathologies, including gingivitis/periodontal disease, aphthous stomatitis, and oral cancer [5]. In this study, a significant relationship between the presence of* H. pylori* in dental plaque and the gingival clinical presentation was found (Table 2). In 1999, in Great Britain, Riggio and Lennon demonstrated the presence of* H. pylori* in 11/29 (38%) subgingival plaques of patients with chronic periodontitis. They suggested that, in this patient group at least, subgingival plaque may be a reservoir for* H. pylori* infection [46]. In 2012, Agarwal and Jithendra found that 60% of the samples were* H. pylori*-positive by PCR in periodontitis group compared to 15% in without-periodontitis group [47]. In Mexico, there are few reports about this association; De la Garza-Ramos et al. found a relationship between the detection of* H. pylori* in dental plaque and the depth of the periodontal pockets in adults [48].

It is known that certain genotypes of* H. pylori *are related to the severity of gastric pathology. Rudi et al. found that* H. pylori* strains of the* vacA s1* allele and the* cagA* gene are more likely to result in peptic ulcer disease [49]. In Mexico, González-Valencia et al. found that* vacA s1* and* cagA*+ strains were more frequent in adults than in children [50]. Another study, conducted by Martínez-Carrillo et al., confirmed that* vacA s1m1* genotypes are the most common in patients with gastric ulcer and chronic gastritis [51]. However, Garza-Gonzalez et al. found that the most frequent genotype was* s2m2* in* H. pylori* isolates from the northeastern region of Mexico, but the* s1m1* genotype was associated with* cagA*-positive strains (*P* < 0.05) [52]. Our results show that the* H. pylori* strains with the* vacA s1m1 *and* s1m2 *genotypes are predominant in dental plaque from Mexican children without dyspeptic symptoms and agree with most of the ones found in Mexican patients with severe gastric pathology [52, 53].

In another study, the most frequent genotype in the oral cavity and gastric mucosa was* vacA s1bm1 *[54]. Román-Román et al. found that 47/196 (24%) patients were coinfected (saliva and gastric biopsy).* H. pylori vacA s1m1 *or* s1m2* genotypes (either or both) were detected in 41.5% of the patients with chronic gastritis. The* s1m1/s1m2* genotypes, alone or together, were found simultaneously in saliva and gastric biopsy from the same patient [32]. These results and others [33] support our findings; however, as far as we know this is the first study of genotyping* vacA* in oral samples from Mexican children without dyspepsia symptoms. Otherwise, we found that* s1m1/s1m2* genotypes alone or together were mostly detected in participants with mild or moderate gingivitis (Table 4).

People infected with* H. pylori *who have a functional* cag*-PAI have increased mucosal concentrations of IL8, neutrophilic infiltration into the gastric mucosa, and increased risk of developing the gastric ulcer and cancer [29, 55, 56]; however other researchers did not agree to this [31]. Román-Román et al. conducted a study in a population from Southern Mexico, and they observed an association between* H. pylori *and the* s1m1 *genotype with gastric cancer, but* cagA* was not associated with the diagnosis [57]. In our study, the* cagA* status was related to the gingival clinical presentation in* H. pylori*-positive children (*P* < 0.001). The* s1m1*/*cagA*+ or* s1m2*/*cagA*+ combinational genotypes alone or together were mostly detected in dental plaque of children with mild or moderate gingivitis. It is possible that these genotypes play a role in the development of periodontal disease in asymptomatic people with poor oral hygiene measures.

## 5. Conclusions

Our results demonstrate that* H. pylori* oral infection is frequent among asymptomatic Mexican children and that, in one individual, more than one* H. pylori* strain may exist in dental plaque. The prevalence of* H. pylori* and detection of some* vacA*/*cagA* genotypes-associated gastrointestinal disease in the oral cavity could be related to the progression of gingivitis in children without dyspeptic symptoms.

## Figures and Tables

**Table 1:** PCR primers and probes used for detection and genotyping of *H. pylori*.

Gen	5′-3′ sequence	Product size (bp)	Reference
16S rRNA	F: GATAGTCAGTCAGGTGTGAAATCC	196	In this study
	R: GTTTGCTCCCCACGCTTT		
	Probe: AAAATCCGTAGAGATCAAGAGGA		
*cagA*	F: GACCGACTCGATCAAATAGCA	113	
	R: TTAGCTGAAAGCCCTACCTTAC		
	Probe: AGTGGTTTGGGTGATGTAGGGCAA		

*vacA s*	vacA1F: CTGGT(C/T)TAAAGTCGCACCCTTTGTGC	*s1*: 120 *s2*: 150	Koehler et al. [25]
	vacA1R: CAATGGCTGGAATGATCACGGTTGT(A/G)		
	vacA2F: CAAACACACCGCAAAATCAATCGCCC		
*vacA m1*	m1F1: CAACAATCAAGGCACTATCAA(C/T)TA	301	
	m1R1: CCGCATGCTTTAATGTCATCAG		
	m1F2: TGGTCCGAGGCGGG(A/C)AAGT		
	m1R2: TCATCAGTATTTCGCACCACAC		
*vacA m2*	m2F1: TTTGGAGC(C/T)CCAGGAAACATTG	102	
	m2R1: C(C/T)ACACGCCCATCTTGGACAA		
	m2F2: ACCCTAAA(C/T)AGCAACGCAAGC		
	m2R2: GACAAAAAGATTCATCGTGCCTT		

**Table 2:** Prevalence of *H. pylori* in dental plaque from Mexican children by gender and age and the gingival clinical presentation.

	Total *N* = 100	*H. pylori* 16S rRNA gene		*P* value
		Positive *N* = 38	Negative *N* = 52	
*Gender n (%)*				
Female	50 (50.0)	17 (44.7)	33 (53.2)	0.41^a^
Male	50 (50.0)	21 (55.3)	29(46.8)	
*Age*				
Mean (±SD)	8.95 (±3.72)	8.37 (±4.17)	9.31 (±3.4)	0.2425^b^
*Age groups n (%)*				
3–5	21 (21.0)	12 (31.6)	9 (14.5)	0.084^a^
6–8	33 (33.0)	10 (26.3)	23 (37.1)	
9–11	14 (14.0)	2 (5.3)	12 (19.4)	
12–14	26 (26.0)	12 (31.6)	14 (22.6)	
15-16	6 (6.0)	2 (5.3)	4 (6.5)	
*MGI n (%)*				
No inflammation	59 (59.0)	6 (15.8)	53 (85.5)	<0.001^c^
Mild inflammation	35 (35.0)	26 (68.4)	9 (14.5)	
Moderate inflammation	6 (6.0)	6 (15.8)	0	

^a^Chi-square test. ^b^Student's  *t* test. ^c^Exact Fisher test. MGI: modified gingival index.

**Table 3:** Distribution of the gingival clinical presentation by gender and age.

	Modified gingival index			*P* value
	No inflammation *N* = 59	Mild inflammation *N* = 35	Moderate inflammation *N* = 6	
*Gender n (%)*				
Female	32 (54.2)	13 (37.1)	5 (83.3)	0.078^a^
Male	27 (45.8)	22 (62.1)	1 (16.7)	
*Age *				
Mean (±SD)	9.47 (±3.42)	8.77 (±4.02)	4.83 (±2.04)	0.012^b^
*Age groups n (%)*				
3–5	8 (13.6)	10 (28.6)	3 (50.0)	0.135^a^
6–8	22 (37.3)	8 (22.9)	3 (50.0)	
9–11	11 (18.6)	3 (8.6)	0	
12–14	14 (23.7)	12 (34.3)	0	
15-16	4 (6.8)	2 (5.7)	0	

^a^Exact Fisher test. ^b^One-way ANOVA test.

**Table 4:** Distribution of *vacA* genotypes, *cagA* status, and combinational *vacA*/*cagA* genotypes in dental plaque from *H. pylori*-positive children with gingival clinical presentation.

	Total *N* = 38	*H. pylori-*positive		
		No inflammation *N* = 6	Mild inflammation *N* = 26	Moderate inflammation *N* = 6
^a^ *vacA alleles n* (%)				
*s1*	31 (81.6)	3 (50.0)	22 (84.6)	6 (100.0)
*s2*	10 (26.3)	3 (50.0)	6 (23.1)	1 (16.7)
*m1*	24 (63.2)	4 (66.7)	15 (57.7)	5 (83.3)
*m2*	16 (42.1)	1 (16.7)	13 (50.0)	2 (33.3)
*m0*	3 (7.9)	1 (16.7)	2 (7.7)	0
*vacA genotypes*				
*s1m1*	10 (26.3)	1 (16.7)	6 (23.1)	3 (50.0)
*s1m2*	11 (28.9)	1 (16.7)	9 (34.6)	1 (16.7)
*s2m1*	6 (15.8)	3 (50.0)	3 (11.5)	0
*s1m0*	2 (5.3)	1 (16.7)	1 (3.8)	0
*s2m0*	1 (2.6)	0	1 (3.8)	0
^b^*s1m1/s1m2*	5 (13.2)	0	4 (15.4)	1 (16.7)
^b^*s1m1/s2m1*	3 (7.9)	0	2 (7.7)	1 (16.7)
*cagA status*				
Positive	27 (71.1)	1 (16.7)	21 (80.8)	5 (83.3)
Negative	11 (28.9)	5 (83.3)	5 (19.2)	1 (16.7)
*vacA/cagA genotypes*				
*s1m1/cagA*+	9 (23.7)	1 (16.7)	5 (19.2)	3 (50.0)
*s1m1/cagA*−	1 (2.6)	0	1 (3.8)	0
*s1m2/cagA*+	5 (13.2)	0	5 (19.2)	0
*s1m2/cagA*−	5 (13.2)	1 (16.7)	3 (11.5)	1 (16.7)
*s2m1/cagA*+	4 (10.5)	0	4 (15.4)	0
*s2m1/cagA*−	3 (7.9)	3 (50.0)	0	0
*s1m1/s1m2/cagA*+	4 (10.5)	0	3 (11.5)	1 (16.7)
*s1m1/s2m1/cagA*+	3 (7.9)	0	2 (7.7)	1 (16.7)
*s1m1/s2m1/cagA*−	1 (2.6)	0	1 (3.8)	0
^c^Undetermined	3 (7.9)	1 (16.7)	2 (7.7)	0

^a^Frequency and percentages based on *H. pylori*-positive patients. ^b^Coexistence of genotypes; ^c^*vacA* genotypes with *m0* alleles; *m0*: nontypeable for middle region.
